# Successful treatment with Omalizumab of a child affected by Systemic Mastocytosis: clinical and biological implications

**DOI:** 10.1186/s13052-022-01402-7

**Published:** 2023-01-13

**Authors:** Grazia Bossi, Valeria Brazzelli, Mara De Amici, Daniela Pietra, Chiara Raviola, Matteo Naso, Corrado Regalbuto, Federica Boselli, Valeria Fortina, Gian Luigi Marseglia

**Affiliations:** 1 Department of Pediatrics, Fondazione Istituto di Ricovero e Cura a Carattere Scientifico (IRCCS) Policlinico San Matteo, Viale Golgi n.19, 27100 Pavia, Italy; 2 Institute of Dermatology, Fondazione Istituto di Ricovero e Cura a Carattere Scientifico (IRCCS) Policlinico San Matteo, Pavia, Italy; 3 Immuno-Allergology Laboratory, Clinical Chemistry Unit, Fondazione Istituto di Ricovero e Cura a Carattere Scientifico (IRCCS) Policlinico San Matteo, Pavia, Italy; 4 Department of Hematology Oncology, Fondazione Istituto di Ricovero e Cura a Carattere Scientifico (IRCCS) Policlinico San Matteo, Pavia, Italy; 5 Pediatric School of Specialization, University of Pavia, Pavia, Italy; 6 Department of Clinical-Surgical Diagnostic and Pediatric Sciences, University of Pavia, Pavia, Italy

**Keywords:** Systemic Mastocytosis, Children, Omalizumab, Tryptase, Total IgE

## Abstract

**Background:**

Pediatric Mastocytosis is a rare and heterogeneous disease, characterized by accumulation of mast cells in the skin (Cutaneous Mastocytosis) and/or, less frequently, in other organs, mainly liver, spleen, bone marrow, lymph nodes and gastrointestinal tract (Systemic Mastocytosis). Patients affected by Systemic Mastocytosis show symptoms caused by  a massive release of mast cell mediators: itching, flushing, abdominal pain, generalized weakness, fatigue and neuropsychiatric disorders. Moreover, children with Systemic Mastocytosis are at greater risk of anaphylactic/anaphylactoid reactions, often poorly controlled by the conventional therapy with antihistamines, mast cells stabilizers and steroids. As a result, children affected by Systemic Mastocytosis have a poor quality of life and suffer the consequence of prolonged steroidal treatment.

**Case presentation:**

A child with Systemic Mastocytosis and severe symptoms, refractory to symptomatic and steroidal therapy, has been successfully treated with Omalizumab, an anti-IgE monoclonal antibody usually employed in allergic patients with severe asthma and orticaria. The onset of clinical benefit of Omalizumab therapy was extraordinarily rapid, but proved to be strictly dependent on drug administration. The child has become completely and steadily asymptomatic. No other anaphylactic episodes have been reported. Steroid treatment could be definitively withdrawn after the second dose of Omalizumab, and all the other medications were later reduced. Twenty months after beginning, Omalizumab therapy is still ongoing with good symptomatology control; no side effects have been observed so far.

**Conclusions:**

In our experience, Omalizumab is an effective treatment for children affected by Systemic Mastocytosis not responding to conventional medical treatments. The main strengths of this therapy are its rapid and extraordinary efficacy to control the severe mast cells mediator-related symptoms, the lack of side effects and its steroid-sparing effect. However, more extensive and controlled studies in pediatric patients affected by Systemic Mastocytosis are needed to substantiate these promising findings.

## Background

Mastocytosis is a rare disease, with a prevalence of 1/10.000, characterized by clonal expansion and accumulation of mast cells in different tissues, mainly skin, and various organs, such as bone marrow, gastrointestinal tract, liver, spleen and lymph nodes [1].

Although the advent of new diagnostic and prognostic tools have posed the need for some refinements, the most recent World Health Organization (WHO) classification is still valid [1, 2]. Accordingly, three major types of Mastocytosis are recognized: Cutaneous Mastocytosis (CM), Systemic Mastocytosis (SM) and localized mast cell tumors [3].

In CM, mast cell proliferation is limited to the skin [4], while mast cell infiltration of extracutaneous organs, with or without skin involvement, is the distinctive feature of SM, which can vary in severity, ranging from the indolent/smoldering form to the aggressive one, eventually associated with haematological neoplasia [3].

Children are mostly affected by one of the three types of CM: isolated skin mastocytoma, maculopapular-CM (further characterized by two subvariants: monomorphic or polymorphic) and diffuse-CM. Pediatric CM usually resolves spontaneously before puberty onset, but persistent CM is described [5]. SM is usually a disease of adulthood, but, although rarely, also children can be affected [6].

Several somatic mutations in the *c-KIT* gene have been linked to the development of SM: the most common of these genetic anomalies is the D816V mutation, that produces enhanced survival and proliferation of mast cells [7].

Patients affected by SM report symptoms caused by the massive release of mast cell mediators (itching, flushing, abdominal pain, generalized weakness, fatigue and neuropsychiatric manifestations, such as headache, depression, anxiety, irritability, lack of concentration), and have an increased risk of anaphylactic/anaphylactoid reactions. The triggers for mast cells activation can be either allergic and IgE-mediated (food, drugs, Hymenoptera venom) or IgE-independent (drugs, physical factors, stress, extreme temperatures, infections) [1, 8, 9].

Usually symptoms of SM respond to treatment with antihistamines, mast cells stabilizers (as montelukast and cromolyn sodium) and corticosteroids [10], but often these therapies are ineffective. In particular, pediatric patients are at great risk of severe, short and long-term side effects related to steroid treatment.

Omalizumab, a recombinant humanized monoclonal antibody against IgE approved for allergic asthma [11, 12] and chronic spontaneous urticaria [13], has been successfully used in patients affected by systemic or cutaneous mastocytosis [14–17]. Most of the patients treated with Omalizumab reported in literature were adults [17–31], while the experience in children is very limited [32–35].

Hereby we report the case of a patient affected by SM, refractory to traditional medications, treated with Omalizumab, who obtained complete symptoms remission and a significant improvement in quality of life.

## Case presentation

A 8-year-old caucasian male was referred to our center after an episode of anaphylaxis of unknown origin (hypotension, tachycardia, general flushing, but no urticaria or angioedema) and persistent high tryptase value (27.5 ng/ml; n.v. < 11.4).

His past medical history was remarkable for the appearance of hyperpigmented skin lesions from the second month of age and the further diagnosis of CM (Urticaria Pigmentosa) at the age of 12 months, confirmed by skin biopsy. The skin lesions had started to regress when the child was 5 years old. The next medical history had been uneventful, except for rare occurrence of flushing, gastrointestinal discomfort and bronchospasm, with spontaneous resolution and without any effect on the quality of life.

When the child first came to our attention, his physical exam was normal; in particular, no skin lesions suggestive of CM could be observed. The working diagnosis was evolution of CM towards the systemic form of the disease, and the patient underwent an extensive diagnostic workup, including, among others, bone marrow biopsy, chest radiography, abdominal ultrasound and bone density scan. SM was verified by one major criterion and two minor WHO criteria: presence of dense aggregates of mast cells in bone biopsy (> 15), localized especially in the paratrabecular foci, confirmed by tryptase immunochemistry and expression of CD2, CD25 and CD117 (major criterion); persistently elevated serum tryptase plus evidence of D816V point mutation in the *c-KIT* gene in bone marrow biopsy (minor criteria). The *c-KIT* mutation was detected also in peripheral blood. Absence of B- and C-findings led to the diagnosis of indolent SM. Over time, our patient experienced frequent disease flares, characterized by recurrent (daily or almost daily) outbreaks of flushing, gastro-intestinal complaints (mainly diarrhea, abdominal pain), palpitations, musculoskeletal symptoms, fatigue. In the meanwhile, serum tryptase values kept on increasing up to 44 ng/ml.

The child was treated unsuccessfully with high doses of oral non-sedating antihistamines (cetirizine 10 mg up to 2 times daily, plus ketotifen 1 mg twice daily), cromolyn sodium (250 mg, 4 times daily) and topical steroids. Oral steroids (prednisone 1-2 mg/kg.day for average 2-5 consecutive days), firstly on demand and then on a daily basis, added only side effects without improving symptomatology. The child’s quality of life continued to deteriorate, with many lost school days, need to stop sporting activities and consequent social withdrawal.

One year after the SM diagnosis, the first line therapies failure was evident and we decided to treat our patient with Omalizumab (Xolair®; Genentech, San Francisco, CA, USA) on compassionate use, keeping unchanged the current therapy. Omalizumab was administered subcutaneously every 4 weeks and the dose of 300 mg was calculated according to the patient’s weight. Amazingly, the child became totally asymptomatic already after the second dose of Omalizumab.

To better objectivate the response to therapy, a modified Mastocytosis Symptom Assessment Form (MSAF) was carried out prior to each injection [35, 36]. In this score, every day in a week, each symptom was scored on a scale from 0 to 10. Before starting the Omalizumab therapy, the reported score ranged from 7/10 to 10/10 for all the symptoms. Already after the first dose of Omalizumab, the MSAF score decreased significantly, reached 0 after the third dose and remained unchanged for the subsequent months.

When, due to the COVID-19 quarantine, a dose of Omalizumab was missed, after 6 weeks after the last treatment, the patient returned to be symptomatic (flushing, headache, diarrhea) and steroid-dependent. Short after the reintroduction of Omalizumab therapy, the symptoms completely disappeared and, since then, steroids could be permanently discontinued. No other anaphylactic epysodes were observed.

Omalizumab has been well tolerated, without side effects, except for minimal local swelling. Cetirizine treatment was gradually reduced and permanently interrupted in  nine months.

The patient had been strictly monitored, but we did not notice any changes in routine blood analyses, including hemoglobin, leukocyte and differential count, platelets, liver and kidney function test or others metabolic parameters (data not shown).

Serum tryptase levels remained stable (median value 35,1 μg/L and 38,8 μg/L, before and during Omalizumab respectively).

**Fig. 1:**
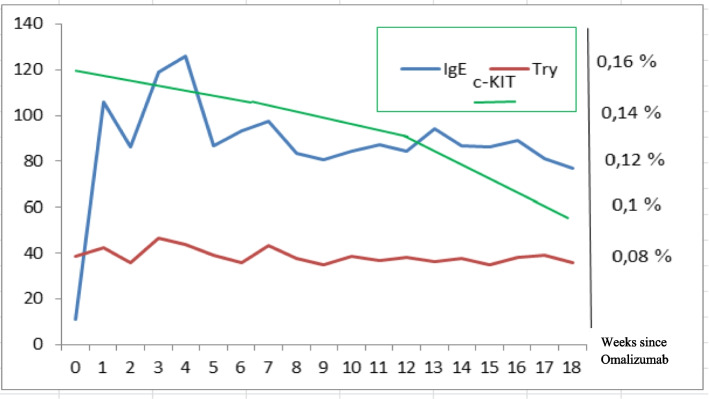
Biological effects (total Ig-E, serum tryptase and c-Kit 816 allele burden in peripheral blood) of Omalizumab therapy. Legend: Try = tryptase

Total IgE levels, normal at baseline (11 KU/l), increased by 10-fold after the first dose of Omalizumab and thereafter, despite a slight decrease, remained steadily increased (7.5-fold) (Fig. 1). The *c-KIT* D816V allele burden in peripheral blood decreased by about 40% (from 0.1598 to 0.097%).

Twenty months later, Omalizumab therapy is still ongoing. The adjuvant treatment (ketotifen 1 mg/day, cromolyn sodium 250 mg × 3 daily), although much reduced, has been maintained, with the program to further reduce it over time. The patient is totally symptom-free, without disease flares; MSAF score is steadily 0. The child’s static-weight growth is adequate, the school attendance returned to be regular and a moderate sporting activity has been resumed.

In view of its extraordinary effectiveness, we did not plan if, when and how to discontinue Omalizumab. In alternative, we chose to prolong the treatment interval, which is currently 5 weeks. With the aim to contain the costs and to maintain the actual good compliance of the patients and his family, we are going to gradually increase the treatment interval, reaching the maximum interval length adequate to provide the symptoms control.

## Discussion and conclusions

Omalizumab is a recombinant humanized murine monoclonal IgG1 antibody, that reversibly binds the free serum IgE at the same site that IgE would bind to its high-affinity receptor (FcɛRI) on the surface of mast cells and basophils. By this mechanism, Omalizumab not only reduces free IgE in the serum, but also, depleting free IgE, strongly downregulates the mast cells and their mediators release [37, 38].

In Italy, Omalizumab is currently approved for adults, adolescents (> 12 years) and children (6-12 years), affected by some IgE-mediated chronic diseases, in particular severe, unresponsive allergic asthma and chronic urticaria. Nevertheless, off-label successful use of Omalizumab has been reported for other different conditions, definitely mast cells-mediated: drug allergies [39], idiopatic angioedema [40], and different mast cell disorders (SM, CM and idiopathic mast cells activation syndrome) [29].

In literature there is only one randomized, placebo-controlled study on the effect of Omalizumab in adult patients with SM. Due to some limitations of this study, no statistical significant difference among treated and untreated patients could be detected, but all the considered symptoms showed a compelling trend toward improvement [31]. Besides that, 15 previous case reports and 3 case series have reported successful treatment of both SM and CM with Omalizumab (Table 1).

**Table 1:** Studies on patients with mastocytosis treated with Omalizumab

Study	PTs (n)	Age (yr)	Dose	Therapy lenght	Total IgE	Tryptase ng/ml
***Systemic Mastocytosis***						
Carter et al., 2007 [17]	2	17 /51	300 mg/4 wk	5.5/4 yrs	↓	=
Pitt et al., 2010 [32]^a^	1	15	300 mg/4 wk	14 mos	↓	=
Kontou-Fili et al., 2010 [19]	1	48	300 mg/4 wk.	2 yrs	na	↓
Douglass et al., 2010 [31]	1	54	150 mg/4 wk	14 mos	na	↓
Molderings et al., 2011 [21]	2	48/ 53	150 mg/4 wk.	4.5/6 mos	na	↓
Kibsgaard et al., 2014 [23]	1	31	300 mg/4 wk	15 mos	na	=
Lieberoth et al., 2015 [25]	2	48/57	300 mg/4 wk.	11/15 mos	na	na
Constantine et al., 2018 [26]	2	29/62	300 mg/4 wk	12 yrs	↑	=/↓
Broesby-Olsen et al.,2018 [27]	11	26-48	75-300 mg/2-4 wk	1-73 mos	na	=
Slapnicar et al., 2019 [28]	6	38-75	300 mg/2-4 wk.	3mos/2 yrs	=	↓/↑
Lemal et al., 2019 [29]	29	17-93	300 mg/2-4 wk	2-40 mos	↑	=
Distler et al., 2020 [31]	5	24-67	150 mg/4wk	6 mos	na	=
***Cutaneous Mastocytosis***						
Siebenhaar et al., 2007 [18]	1	56	150 mg/2-4 wk	12 mos	na	na
Bell et al., 2012 [33]^a^	1	11	150 mg/4 wk	10 mos	na	na
Matito et al., 2013 [34]^a^	1	12	450 mg/4 wk	3 mos	na	na
Paraskevopoulos et al., 2013 [22]	1	25	300 mg/4-7 wk	2 yrs	na	na
Sokol et al., 2014 [24]	1	77	375 mg/4 wks	12 mos	na	↓
Hughes et al., 2018 [35]^a^	2	2	150 mg/4 wk	3 mos	na	=
Hinnojosa et al., 2019 [30]	2	51/36	300 mg/4 wk	7/5 mos	na	na
Lemal et al., 2019 [29]	11	17-93	300 mg/4 wk	2-40 mos	↑	=

*na* not assessed

^a^Studies on pediatric patients

The main positive effect of Omalizumab in adult patients with SM was the prevention of recurrent episodes of anaphylaxis [27], but also other symptoms, such as gastrointestinal complaints, asthenia, pruritus and flushing were positively affected. Patients with CM experienced reduction of skin symptoms. In all the  reported cases, the patients improved quickly and no side effects were observed.

It is important to emphasize, however, that only 5/81 (6%) patients reported in the literature were under the age of 18 [32–35], and only one out of the 5 children described was affected by SM [32].

As far as we know, ours is the second pediatric case of SM successfully treated with Omalizumab.

In our patient the clinical improvement was very rapid and complete. Omalizumab was well tolerated and no adverse effects were observed. Twenty months after beginning Omalizumab treatment, our patient is symptom free, without symptoms flares. No other anaphylactic episodes were observed. Steroid therapy was definitely stopped and the antihistamines have been steadily reduced.

The clinical benefit proved to be strictly dependent on timely Omalizumab administration every 4 or 5 weeks at the most, and, unlike what reported elsewhere for a child with CM [34], in our patient Omalizumab treatment could not be stopped so far. Actually, in our experience, the Omalizumab clinical positive effect time length perfectly overlaps its pharmacokinetics, because the complexes resulting from the binding between Omalizumab and the Fc portion of the IgE are cleared with a terminal half-life of 26 days [41].

In line with most of the previous case reports of pediatric patients affected by SM [32] or CM [35], serum tryptase levels of our patient did not significantly change during Omalizumab treatment. This finding seems to support the hypothesis that Omalizumab efficacy mainly depends on a different mast cells mediator release process and the consequent decrease in their activation state, rather than on mast cells number or degranulation [38].

According to what usually observed in allergic patients during Omalizumab treatment, our patient showed a significant and progressive increase in total IgE levels, already evident after the first doses. This finding, reported also in patients affected by different types of mast cell disorders treated with Omalizumab [29], is otherwise in contrast with the results of the adolescent with SM described elsewhere [32]. The increase in total IgE levels (including free and Omalizumab-bound fractions) could be explained by the effect of stabilization of total IgE induced by Omalizumab, or as the result of the of the longer biological half-life of serum IgE [42].

Anyway, in our case, the relationship between the IgE increase induced by Omalizumab and the clinical symptoms remission is unquestionable. This evidence could support the hypothesis that most of the symptoms of patients affected by SM could be Ig-E mediated, or, in the alternative, that the breaking of the chemical bond between IgE and mast-cells could somehow interfere with the mast cells mediators release or their pathological network.

As far as the other biological effects of Omalizumab treatment, despite the symptoms remission, the tryptase levels remained elevated, while, on the contrary, we observed a strict relationship between the clinical improvement and the significant reduction of the *c-KIT* D816V allele burden, that could be regarded as a good biomarker for the biological monitoring of patients with SM treated with Omalizumab or any other future biological modifier.

In conclusion, this case highlights some relevant key points regarding Omalizumab treatment of pediatric patients affected by SM not responding to conventional medical treatments: its rapid and extraordinary efficacy to control the severe mast cells mediator-related symptoms, and its steroid-sparing effect.

However, further studies and, likely, more patients are required to fully understand the mechanisms of action of Omalizumab in mast cell disorders, especially in pediatric patients. In particular, it is conceivable that, just starting from the evidence of the efficacy of Omalizumab in controlling the mast cell activation and the consequent mediators release, some new insights in biological pathological mechanisms of SM could be better understood. A better knowledge of SM pathogenesis could, in turn, open new paths for a treatment able to definitively cure SM and not only to control its symptoms.

## Data Availability

The dataset used and/or analysed for the current case report are available from the corresponding author on reasonable request.

## Abbreviations

WHO: World Health Organization
CM: Cutaneous Mastocytosis
SM: Systemic Mastocytosis
MSAF: Mastocytosis Symptom Assessment Form
